# Mechanism of genistein regulating the differentiation of vascular smooth muscle cells into osteoblasts via the OPG/RANKL pathway

**DOI:** 10.18632/oncotarget.20167

**Published:** 2017-08-10

**Authors:** Cheng Shen, Ye Yuan, Fuping Li, Yijie Hu, Yi Song, Shulin Zhao, Qianjin Zhong

**Affiliations:** ^1^ Department of Cardiovascular Surgery, Institute of Surgery Research, Daping Hospital, Third Military Medical University, Chongqing, China

**Keywords:** genistein, OPG/RANKL pathway, VSMCs

## Abstract

**Objective:**

The present study aimed to investigate the mechanism of genistein, a tyrosine kinase inhibitor, regulating the differentiation of vascular smooth muscle cells (VSMCs) into osteoblasts via the OPG/RANKL (Osteoprotegerin/Receptor Activator of Nuclear Factor-κB Ligand) pathway.

**Methods:**

The mouse VSMCs were isolated, purified and cultured. We constructed the LV5-Tnfrsf11b overexpression lentiviral vector and LV3-OPG-309 interference lentiviral vector. The OPG overexpression was induced and the growth of VSMCs infected with the lentiviral vector was observed. The VSMC calcification and control group were treated with different doses of genistein. The mRNA and protein expression levels of OPG, α-SM-actin (smooth muscle actin), ALP (alkaline phosphatase) and OPN (osteopontin) were detected in VSMCs after treatment using RT-PCR and Western Blot.

**Result:**

We induced OPG overexpression and performed lentiviral vector infection of the VSMCs to suppress OPG expression, respectively, which was followed by treatment with genistein. The results showed that the relative expression of OPG was the highest in the VSMC calcification +genistein +OPG overexpression-inducing treatment group. It was the lowest in the VSMC calcification +OPG expression-suppressing treatment group. The relative expression of ALP was the highest in the VSMC calcification +OPG expression-suppressing treatment group, and the lowest in the VSMCs+genistein treatment group.

**Conclusion:**

OPG gene plays an important regulatory role in the growth of VSMCs, by suppressing the calcification of VSMCs. Genistein could regulate the differentiation of VSMCs into osteoblasts via the OPG/RANKL pathway in a dose-dependent manner.

## INTRODUCTION

Vascular calcification is a common pathological process shared by atherosclerosis, diabetes, chronic kidney diseases and aging [1]. Directly contributing to the occurrence and progression of cardiovascular diseases, vascular calcification usually manifests as decreased compliance of vascular walls and increased hardness of vascular walls, and it may easily lead to myocardial ischemia, left ventricular hypertrophy and heart failure [2]. Vascular calcification is also one of the major reasons of thrombosis, plaque rupture and high incidence and high mortality of cardiovascular and cerebrovascular diseases. Study shows that most patients of coronary artery disease are combined with vascular calcification [3]. Conditions such as dyslipidemia, hypertension, diabetes and kidney failure will all promote arterial calcification [3–5]. Therefore, a thorough understanding on the pathogenesis of vascular calcification is crucial for the prevention and treatment of vascular calcification.

Vascular calcification is considered as an active regulatory process resembling bone mineralization, where VSMCs play an extremely important role [6]. Phenotypic transition of VSMCs into osteoblasts is among the major reasons of vascular calcification. This process involves the metabolism of calcium and phosphorus, formation of osteoblasts, and action of inhibitory factors and growth factors [7]. OPG/RANKL pathway, a newly discovered signaling pathway related to the interaction between osteoblasts and osteoclasts, is found to be relevant to vascular calcification [8–9]. Current studies on the mechanism of vascular calcification generally focus on calcium and phosphorus metabolism and the promoting and inhibitory factors of calcification [10–11]. However, the pathogenesis of vascular calcification and the related regulatory mechanism are rarely reported.

Genistein [49,5,7-trihydroxyisoflavone or 5,7-dihydroxy-3-(4-hydroxyphenyl) chromen-4-one] (C15H10O5) belongs to a multifunctional natural iso-flavonoid class of flavonoids with a 15-carbon skeleton. It was isolated for the first time from Genista tinctoria L. in 1899 and named after the genus of this plant. Genistein was reported to be associated with cell proliferation and tumor pathology [12–13]. Furthermore, genistein also has been reported to be involved in bone metabolism and osteoporosis [14]. However, the mechanism of genistein regulating VSMCs remain unclear. In the present study, we explored the mechanism of genistein regulating VSMCs via the OPG/RANKL pathway.

## RESULTS

### *In vitro* culture of VSMCs and construction of overexpression and interference lentiviral vectors

**Figure 1:**
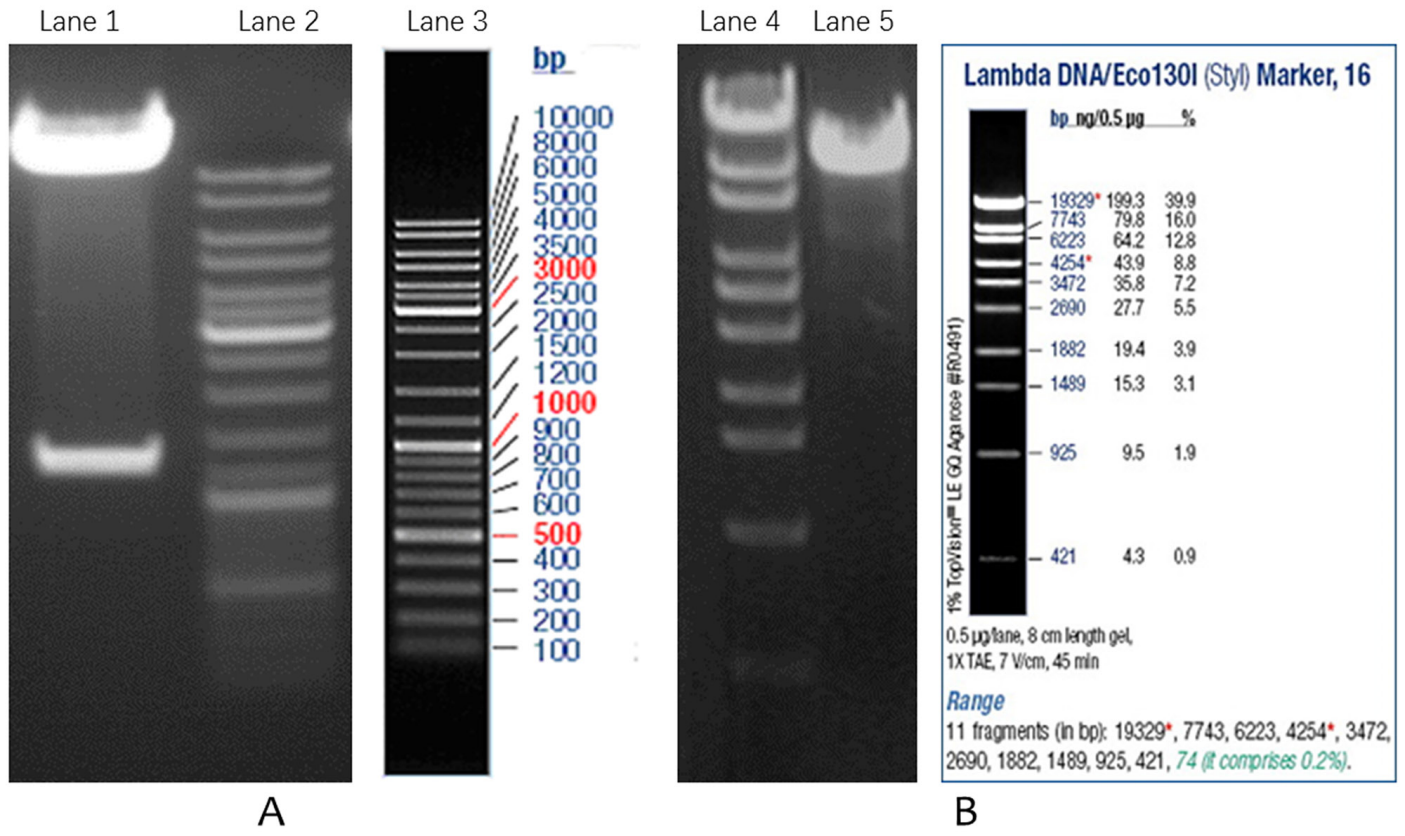
Verification of overexpression and interference lentiviral vectors **(A)** Double digestion of LV5-Tnfrsf11b mus recombinant plasmid; **(B)** Double digestion of LV3-OPG-309 recombinant plasmid. Lane1 is recombinant plasmid, Lane 2 and 3 are DNA markers, Lane 4 is DNA marker, Lane5 is recombinant plasmid.

Mouse VSMCs were cultured *in vitro* and showed good growth. The LV5-Tnfrsf11b mus overexpression and LV3-OPG-309 interference lentiviral vectors were verified by double digestion and sequencing, as shown in Figure 1A and 1B. Viral titer determination indicated that at 72h post-transfection, the titer of Tnfrsf11b mus overexpression vector was 1×10^8^TU/mL, the titer of LV5NC negative control was 5×10^8^ TU/mL.

### Infection of VSMCs with OPG overexpression and interference lentiviral vectors

**Figure 2:**
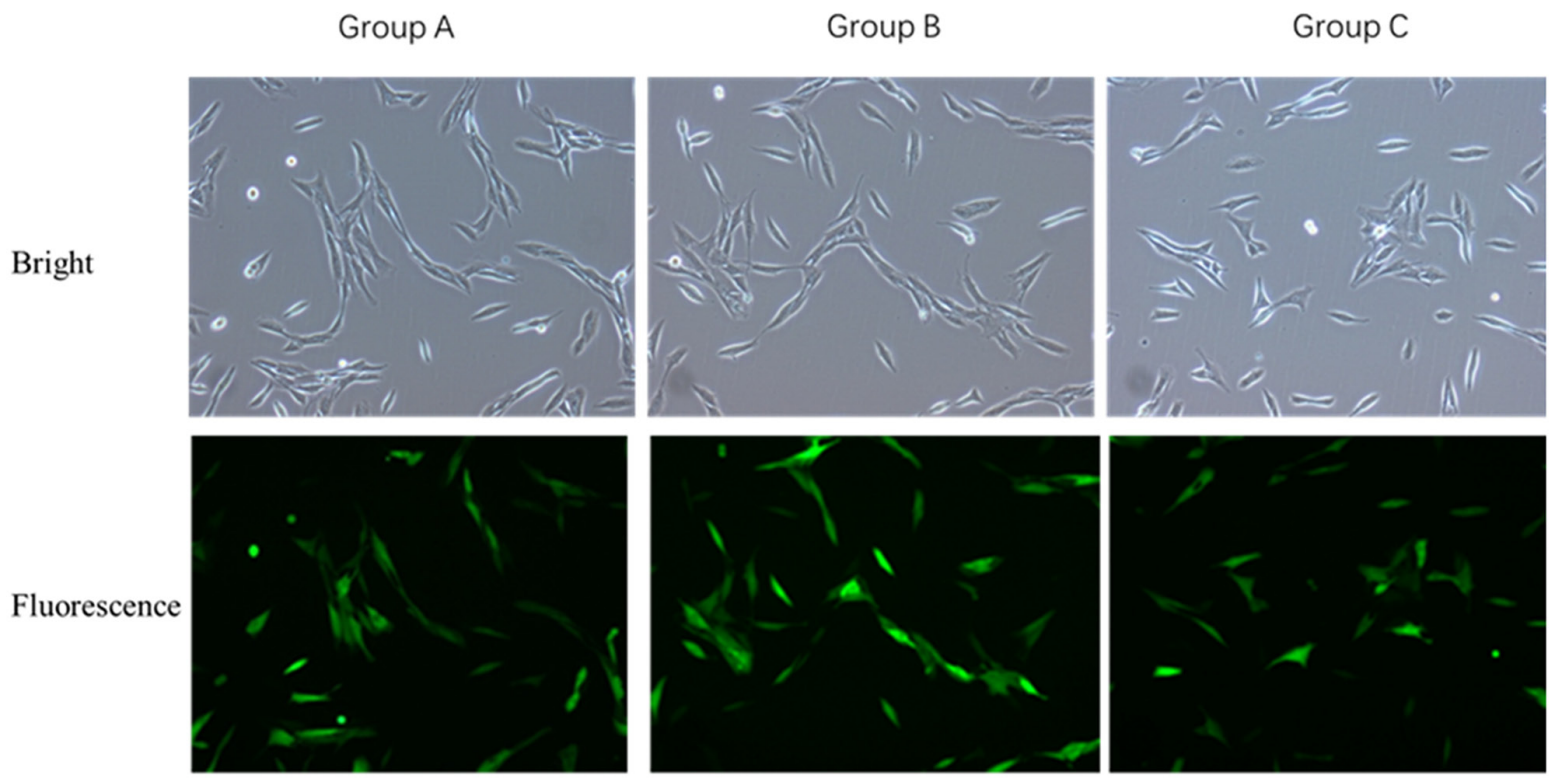
Comparison of VSMCs transfected with OPG overexpression and interference lentiviral vectors **(A)** Control group; Group B: Tnfrsf11b mus overexpression group; **(C)** LV3-OPG-mus-309 interference group.

The VSMCs were infected with OPG overexpression and interference lentiviral vectors, respectively. As observed under the optical and fluorescence microscopes, there was no significant difference in the proliferation of VSMCs infected with OPG overexpression lentiviral vector as compared with the negative control; rather, there were more infected VSMCs than that in the control cells. However, there was a reduction in the VSMCs infected with OPG interference lentiviral vector as compared with the control cells (Figure 2).

### mRNA and protein expression of OPG, α-SM-actin, ALP and OPN genes in VSMCs

**Figure 3:**
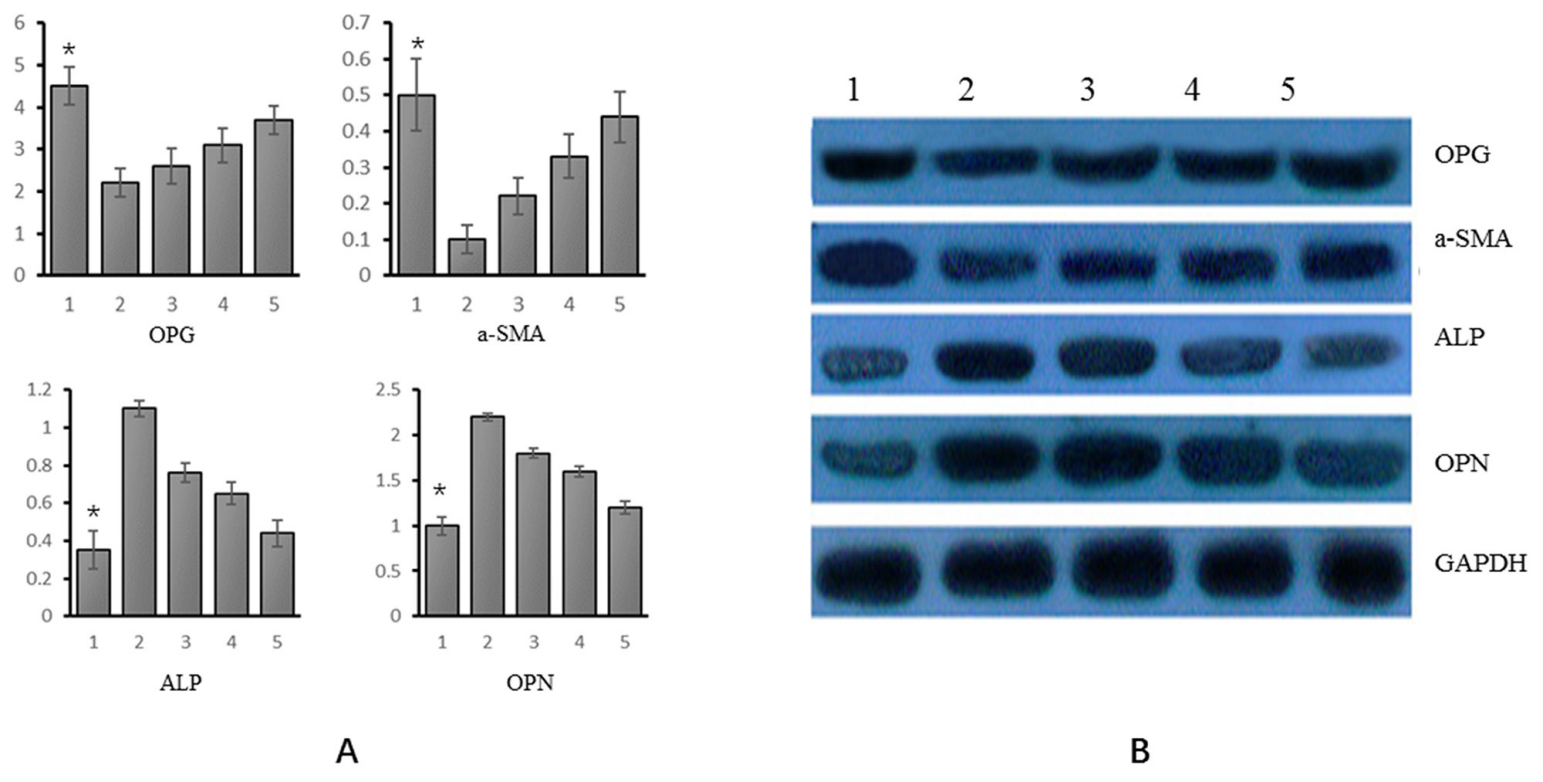
mRNA and protein expression of OPG, α-SM-actin, ALP and OPN in different experimental groups **(A)** mRNA (Group 1: VSMCs+genistein treatmentl; group 2: VSMC calcification; group 3: VSMC calcification+ genistein 10 uM treatment; group 4: VSMC calcification+ genistein 30 uM treatment; group 5: VSMC calcification+genistein 50 uM treatment); **(B)** Protein (Group 1: VSMCs+genistein treatmentl; group 2: VSMC calcification; group 3: VSMC calcification+ genistein 10 uM treatment; group 4: VSMC calcification+ genistein 30 uM treatment; group 5: VSMC calcification+genistein 50 uM treatment). *p<0.05.

Of the 5 experimental groups, the relative expression of OPG was the highest in group 1 and the lowest in group 2. It increased gradually from group 3 to 5. The expression of α-SM-actin and OPG genes varied consistently. Relative expression of ALP was the lowest in group 1 and decreased from experimental group 2 to 5. The expression of OPN and ALP genes varied consistently, as shown in Figure 3A.

As indicated by WB, each of the four genes showed consistent variations of mRNA and protein expression. That is, the relative expression of OPG was the highest in group 1 and it increased from experimental group 2 to 5. The relative expression of α-SM-actin and OPG genes varied consistently. The relative expression of ALP was the lowest in group 1 and it decreased from experimental group 2 to 5; the expression of OPN gene and ALP gene varied consistently, as shown in Figure 3B.

### The VSMCs infected with OPG overexpression and interference lentiviral vectors were then treated with genistein. The changes in mRNA and protein expression of OPG, α-SM-actin, OPN, and ALP genes

**Figure 4:**
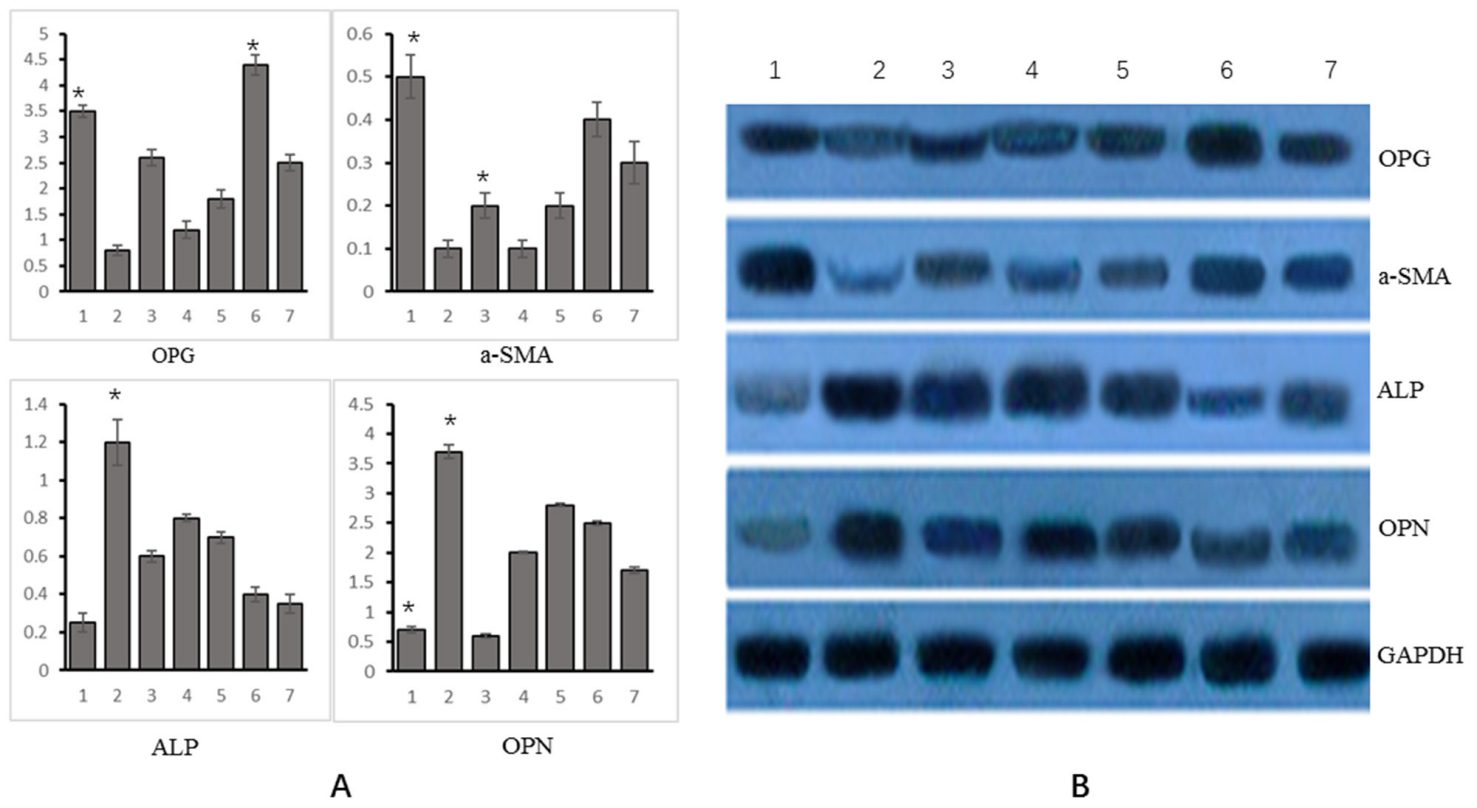
Changes in mRNA and protein expression of OPG, α-SM-actin, ALP, OPN and Runx2 genes in different experimental groups **(A)** mRNA (Group 1: VSMCs+ genistein treatment; group 2: VSMC calcification+OPG interference treatment; group 3: VSMC calcification+OPG overexpression; group 4: VSMC calcification+OPG negative control; group 5: VSMC calcification+genistein treatment+OPG interference; group 6: VSMC calcification+genistein treatment+OPG overexpression treatment; group 7: VSMC calcification+genistein treatment+negative control); **(B)** Protein (Group 1: VSMCs+ genistein treatment; group 2: VSMC calcification+OPG interference treatment; group 3: VSMC calcification+OPG overexpression; group 4: VSMC calcification+OPG negative control; group 5: VSMC calcification+genistein treatment+OPG interference; group 6: VSMC calcification+genistein treatment+OPG overexpression treatment; group 7: VSMC calcification+genistein treatment+negative control.). *p<0.05.

Of 7 experimental groups, the relative expression of OPG was the highest in group 6 and the lowest in group 2. The relative expression of α-SM-actin was the highest in group 1, followed by group 6, and the lowest in group 2. The relative expression of ALP was the highest in group 2 and the lowest in group 1. The expression of OPN and ALP varied consistently. See Figure 4A.

As indicated by WB, the protein expression of OPG, α-SM-actin, ALP and OPN varied consistently with their mRNA expression. The relative expression of OPG was the highest in group 6 and the lowest in group 2. The relative expression of α-SM-actin was the highest in group 1 and the lowest in group 2. The relative expression of ALP was the highest in group 2 and the lowest in group 1. The expression of OPN gene varied consistently with that of ALP gene, as shown in Figure 4B.

## DISCUSSION

In the prsent study, we found OPG gene plays an important regulatory role in the growth of VSMCs, by suppressing the calcification of VSMCs. Genistein could regulate the differentiation of VSMCs into osteoblasts via the OPG/RANKL pathway in a dose-dependent manner.

In essence, vascular calcification is the trans-differentiation of blood vessels into an osteoblastic phenotype and a transformation of vascular tissues into bone tissues [15]. Understanding the mechanism of trans-differentiation will certainly benefit the prevention and reversal of vascular calcification. The current knowledge related to the pathogenesis of vascular calcification highlights calcium and phosphorus metabolism and the role of calcification inhibiting and promoting factors and senescence of VSMCs. It is generally agreed that OPG gene and OPG/RANKL pathway play an important role in vascular calcification [16]. OPG, a member of the tumor necrosis factor receptor superfamily, regulates bone resorption and widely exists in heart, lung and kidney [17–18]. Schoppet et al. [19] confirmed that *in vitro* OPG overexpression inhibited VSMC calcification; the more severe the vascular calcification, the higher the OPG expression would be. OPG also acts as the soluble receptor for RANKL, blocking the action of RANKL and inhibiting the differentiation of osteoclast precursors into mature osteoclasts. According to Abedin et al., [20] OPG/RANKL pathway plays a decisive role in cell differentiation and maturation as well as atherosclerosis and vascular calcification. OPG is also a receptor antagonist for TNF-related apoptosis-inducing ligand, directly binding to the receptors on VSMCs and endothelial cells to induce cell apoptosis and hence suppressing vascular calcification and atherosclerosis. Recent study shows that the OPG/RANKL pathway has limited impact on the progression of vascular calcification [21]. In our study, VSMCs infected with OPG overexpression lentiviral vector proliferated exuberantly. However, those infected with OPG interference lentiviral vector decreased dramatically in number. This can be probably explained by the decline of OPG's ability in inhibiting VSMC apoptosis, which leads to increased apoptosis of VSMCs and vascular calcification. In addition, through the interaction between VSMCs and genistein, it was found that the inhibitor worked via the OPG/RANKL pathway.

Besides the OPG gene, some calcification inhibiting and promoting factors are also involved in vascular calcification. ALP is the marker for osteogenic differentiation and widely present in different human tissues. Any factors that induce osteoblast proliferation and hyperactivity will increase ALP expression. Study shows that ALP plays an important role in vascular calcification [22]. OPN, as a secretory phosphoprotein, is widely found in bones and teeth rich in minerals. It can bind to osteoclast receptors to cause local mineral dissolution and hence inhibit the formation of hydroxyapatite crystals. Yamaguchi et al. [23] showed that OPN directly bound to hydroxyapatite crystals to suppress their enlargement, thus resulting in the suppression of vascular calcification. Senile VSMCs may display an osteoblastic phenotype. The overexpression of ALP, type I collagen in cells will induce the differentiation of osteocytes. This indicates the important role played by the senile VSMCs in vascular calcification [24]. Study shows that Runx-2 is the most important transcriptional factor in osteoblast differentiation, which can induce the expression of bone matrix proteins in early stages of osteoblast differentiation [25]. We also found that the ALP and OPN genes were involved in vascular calcification. But after treatment with genistein, the expression of these genes was altered. The OPG and α-SM-actin expression increased but ALP and OPN expression decreased after geneistein treatment. We also found the changes of these four genes and their related proteins in a dose-dependent manner. Therefore, we believe that vascular calcification is a complex process involving multiple factors, where the OPG/RANKL pathway dominates.

In conclusion, the present study indicated that genistein could regulate the differentiation of VSMCs into osteoblasts via the OPG/RANKL pathway in a dose-dependent manner.

## MATERIALS AND METHODS

### Laboratory animals, reagents and equipment

SPF BALB/C mice aged four weeks were purchased from Laboratory Animal Center of Third Military Medical University. This study was approved by the ethics committee of Daping Hospital, Third Military Medical University. 293T cell line used to package the lentivirus was purchased from Shanghai Institutes for Biological Sciences. Lentiviral vector LV5 and LV3, shuttle vector, and *E. coli* DH5α were purchased from Invitrogen.

The reagents used were as follows: DMEM, fetal bovine serum (Gibco, USA); PBS (Zhongshan Corporation, Beijing); DNA endonuclease, ligase and marker (Fermentas); agarose and DNA gel recovery kit (Tiangen Biotech (Beijing) Co., Ltd.); plasmid midi preparation kit (Axygen Biotechnology (Hangzhou) Limited); primer synthesis, RT reagent, fluorescent quantitative PCR detection reagent and SYBR green I (Western Biotechnology Inc.); primary antibody for internal reference, and secondary antibodies for goat anti-rabbit IgG and goat anti-mouse IgG (Sigma, USA); PVDF membrane (Dupont, USA); enhanced ECL chemiluminescence detection kit (Pierce, USA).

Small benchtop centrifuge (TGL-16G, China); ultra-high-speed and high-speed centrifuges (Beckman); electronic analytical balance (Precisa12A, Switzerland); pipettee (Eppendorf, Germany); electric-heated thermostatic water bath (DK-8D, China); inverted optical microscope (Olympus, Japan). Water insulation type thermostatic incubator (XMTH-142, China). Cell incubator (Shellab, USA); biosafety cabinet (Shanghai Boxun Industry & Commerce Co., Ltd.); pure water filter (Millipore, Germany); PCR instrument (MG96+ Hangzhou LongGene Scientific Instruments Co., Ltd.); real-time fluorescent quantitative PCR instrument (Eppendorf); inverted fluorescence microscope (Leica); vertical electrophoretic transfer equipment, Trans-Blot® SD semi-dry transfer cell and electrophoresis system (Bio-Rad, USA); image analysis system (LabworksTM Analysis Softwar, USA).

### Isolation, purification and culture of mouse VSMCs

The mice were anesthesized by intraperitoneal injection of 2ml 10% chloral hydrate. The chest and abdomen were opened under aseptic conditions to expose the heart. The thoracic and abdominal organs were removed until exposing the aorta, which was wholely dissociated and added with 1ml sterilized PBS. After repeatedly washing with D-Hanks solution, the adipose tissues surrounding the aorta were carefully stripped with tweezers. The envelope was removed until the aorta was smooth and transparent. The aorta was placed into DMEM and cut open vertically using ophthalmic scissors. The intima was scrapped 2-3 times with a knife blade to remove it. After that, the blood vessel was transferred into a 35mm sterile Petri dish containing DMEM supplemented with 3ml of 20% FBS. The blood vessel was cut into 1 mm×1 mm fragments using ophthalmic scissors. The tissue blocks were uniformly inoculated to the bottom of the culture flask and placed at 37°C in a 5%CO_2_ incubator for 4-5h. Then the culture flask was gently turned so that the tissue blocks were completely immersed in the culture medium. When the cells were 80% confluent, they were digested with 0.25% trypsin and cell passage was performed. The supernatant was collected 2h later and inoculated to another culture flask. The non-adherent cells were collected 24h later and centrifuged for 5min. The cells were resuspended and inoculated to a new culture flask. The cells were purified by repeated differential adhesion. The cells of the 5^th^ to 8^th^ generation were used for further experiment.

### Construction of overexpression and interference lentiviral vectors

The Tnfrsf11b mus fragment was obtained by PCR amplification. The target gene Tnfrsf11b mus was cloned to lentiviral vector LV5. Double digestion was performed on the vector using NotI and BamHI at 37°C for 2h. The vector LV5 was recovered using DNA gel recovery kit. The amplified fragment was subjected to recombinant cloning to the linearized vector LV5 using ClonExpress^®^ Entry One Step Cloning Kit. The competent cells were transformed with the ligation product. Plasmid extraction was perfomred for the bacterial liquid using the plasmid mini extraction kit. The fragment sequence was verified by double digestion. The lentiviral shuttle plasmid, helper plasmid, and carrier plasmid were prepared and subjected to high-purity endotoxin-free extraction and then used for co-transfection of the 293T cells. The medium was replaced by complete medium at 12h post-transfection, and the cells were further incubated for 48h. The supernatant containing the lentiviral particles was collected and concentrated into high-titer lentiviral stock. The 293T cells were transfected and the titer was detected by using the double dilution method.

The double-stranded DNA oligo containing the interference sequence (LV3-OPG-309) was synthesized, with its two terminals containing the restriction enzyme cutting sites as sticky ends; it was connected to the enzyme-digested RNA interference vector. The resulting product was transferred to the competent cells, and the newly generated clones were verified by enzyme digestion. The positive clones were picked for sequencing. After sequence alignment, the confirmed positive clones were the RNA interference lentiviral vector targeting the target gene. The lentiviral shuttle plasmid, helper plasmid, and carrier plasmid were prepared and subjected to high-purity endotoxin-free extraction and then used for co-transfection of the 293T cells. The post-transfection procedures of cell culture and viral titer determination were the same as above.

### Infection of VSMCs with OPG overexpression and interference lentiviral vectors

The mouse VSMCs cultured *in vitro* were infected with OPG overexpression and interference lentiviral vectors, respectively. Four groups were set up: LV5NC negative control, Tnfrsf11b mus overexpression group, LV3NC negative control, and LV3-OPG-mus-309 interference group. The infected cells were observed under the optical microscope and inverted fluorescence microscope. The transfection efficiency was estimated.

### RT-PCR detection of mRNA expression of OPG, α-SM-actin, ALP and OPN genes and WB detection of corresponding proteins in VSMCs

Five groups were set up. Group 1: VSMCs+genistein treatmentl; group 2: VSMC calcification; group 3: VSMC calcification+ genistein 10 uM treatment; group 4: VSMC calcification+ genistein 30 uM treatment; group 5: VSMC calcification+genistein 50 uM treatment. Besides calcification, genistein of different concentrations was added as well to incubate the cells for 21 days. When the VSMCs of the fourth to eighth generation were 70% confluent, DMEM (10% fetal bovine serum) was added for the normal group; the calcification medium was added for the calcification treatment; 10uM, 30uM and 50uM genistein was added for the experimental groups, respectively. The medium was replaced every 2 days.

Total RNA extraction was performed using Trizol reagent. Total RNA was reversely transcribed into cDNA using random primers. Fluorescent quantitative PCR was performed using specific primers (Table 1) and SYBR green I dye. The standard curve was ploted to compare the average relative copy number of different target genes with that of the internal reference gene. Thus the ratios were calculated.

**Table 1:** Sequences of primers used for PCR

Gene		Sequences(5′-3′)	Products
OPG	ID: 18383		189bp
	mOPGF	ATTGGCTGAGTGTTTTGGTGG	
	mOPGR	CGCTGCTTTCACAGAGGTCA	
α-SM-actin	ID:11475		179bp
	ma-SMAF	CCCTGAAGAGCATCCGACA	
	ma-SMAR	CTCCAGAGTCCAGCACAATACC	
ALP	ID: 11647		140bp
	ALP F	ATAACGAGATGCCACCAGAGG	
	ALP R	TTCCACATCAGTTCTGTTCTTCG	
OPN	ID: 20750		178bp
	mOPNF	GCAGACACTTTCACTCCAATCG	
	mOPNR	GGGACTCCTTAGACTCACCGC	
Housekeeping gene	m actin f	GAGACCTTCAACACCCCAGC	263bp
	m actin r	ATGTCACGCACGATTTCCC	

The same grouping scheme was used for WB. Total protein extraction was performed by adding lysis buffer. The protein concentration was quantified using the BCA method. The proteins were separated by SDS-PAGE and transferred to the PVDF membrane. Immune reaction was initiated between the proteins or polypeptides immobilized to the PVDF membrane as antigens and the corresponding antibodies; this was followed by an reaction with the labeled secondary antibodies. The proteins encoded by the target genes and separated by electrophoresis were detected by using the substrate development system. The grayscale values of the target bands were analyzed using the UVP gel image processing system LabWorks 4.6. The ratio of grayscale value of target bands to that of the internal reference gene was calculated.

### RT-PCR

RT-PCR was performed to detect the effect of infection with OPG overexpression and interference lentiviral vectors on VSMCs. Then WB detection was performed to detect mRNA expression of OPG, α-SM-actin, OPN, Runx2 and ALP genes after genistein treatment.

Seven groups were set up. Group 1: VSMCs+ genistein treatment; group 2: VSMC calcification+OPG interference treatment; group 3: VSMC calcification+OPG overexpression; group 4: VSMC calcification+OPG negative control; group 5: VSMC calcification+genistein treatment+OPG interference; group 6: VSMC calcification +genistein treatment+OPG overexpression treatment; group 7: VSMC calcification+genistein treatment+negative control.

The same procedures of cell culture and treatment were used. Table 1 shows the primers used for amplification. For Runx2, the upstream primer was mRunx2F CTACCCAGCCACCTTTACCTAC and the downstream primer was mRunx2R GAACTGATAG-GATGCTGACGAAG; the amplification length was 190bp.

The same procedures of WB were used as mentioned above. The ratio of grayscale value of target bands to that of internal reference gene was calculated.

### Statistical process

All statistical analyses were performed using SPSS17.0 software. Qualitative variables were analyzed using the chi-square test, and quantitative variables using t-test or ANOVA. P<0.05 indicated significant difference.
